# Advance in Targeted Immunotherapy for Graft-Versus-Host Disease

**DOI:** 10.3389/fimmu.2018.01087

**Published:** 2018-05-16

**Authors:** Lingling Zhang, Jianhua Yu, Wei Wei

**Affiliations:** ^1^Institute of Clinical Pharmacology, Anhui Medical University, Key Laboratory of Anti-Inflammatory and Immunopharmacology of Education, Ministry of China, Anti-Inflammatory Immune Drugs Collaborative Innovation Center, Hefei, Anhui, China; ^2^Division of Hematology, Department of Internal Medicine, College of Medicine, The Ohio State University, Columbus, OH, United States

**Keywords:** graft-versus-host disease, immunotherapy, immune inhibitors, immune cells, hematopoietic stem cell transplantation

## Abstract

Graft-versus-host disease (GVHD) is a serious and deadly complication of patients, who undergo hematopoietic stem cell transplantation (HSCT). Despite prophylactic treatment with immunosuppressive agents, 20–80% of recipients develop acute GVHD after HSCT. And the incidence rates of chronic GVHD range from 6 to 80%. Standard therapeutic strategies are still lacking, although considerable advances have been gained in knowing of the predisposing factors, pathology, and diagnosis of GVHD. Targeting immune cells, such as regulatory T cells, as well as tolerogenic dendritic cells or mesenchymal stromal cells (MSCs) display considerable benefit in the relief of GVHD through the deletion of alloactivated T cells. Monoclonal antibodies targeting cytokines or signaling molecules have been demonstrated to be beneficial for the prevention of GVHD. However, these remain to be verified in clinical therapy. It is also important and necessary to consider adopting individualized treatment based on GVHD subtypes, pathological mechanisms involved and stages. In the future, it is hoped that the identification of novel therapeutic targets and systematic research strategies may yield novel safe and effective approaches in clinic to improve outcomes of GVHD further. In this article, we reviewed the current advances in targeted immunotherapy for the prevention of GVHD.

## Introduction

Hematopoietic stem cell transplantation (HSCT) is used to treat hematologic malignancies. Conditional on surviving the first 2 years after BMT, 5 years survival generally exceeds 70% (1). Graft-versus-host disease (GVHD) is a serious life-threatening complication in patients undergoing HSCT resulting from donor T lymphocytes activated by host antigen-presenting cells (APCs), and resulting in an inflammatory response and immune system disorders (2–5). GVHD includes two phases of pathological progress, namely acute GVHD (aGVHD) and chronic GVHD (cGVHD). 20–80% of recipients would develop aGVHD after allogeneic hematopoietic cell transplantation. And the incidence rates of cGVHD range from 6 to 80%, depending on risk factors and diagnostic criteria used (1).

Despite considerable advances in knowing of the predisposing factors, pathophysiology, and diagnosis of aGVHD and cGVHD, a standard therapeutic strategy is still lacking (6–8). The American Society of Blood and Marrow Transplantation developed some recommendations for treatment of aGVHD. The standards of care treatments that are used now days include first-line treatment and second-line treatment. Steroid remains the mainstay of first-line treatment in grades II–IV GVHD. Standard treatment with prednisone showed an overall complete response (CR) rate of 48%, while the percentage of steroid-refractory aGVHD (SR-aGVHD) is approximately 50%. Criteria and indications for secondary systemic therapy of aGVHD have not been systematically defined. Secondary systemic therapy may be indicated earlier in patients who cannot tolerate high-dose glucocorticoid treatment. Agents for second-line treatment of aGVHD include immunosuppressive agents (mycophenolate mofetil, pentostatin, cyclophosphamide) and monoclonal antibodies (mAbs), and so on. The use of cyclophosphamide at high doses to prevent GVHD would reduce the cumulative 1-year incidence of cGVHD to 15% or less (9).

Apart from systemic corticosteroids, the therapies targeting immune cells and immune molecular have been applied currently to inhibit GVHD (10–15). Regulatory T cells (Tregs), tolerogenic dendritic cells (TDCs), or mesenchymal stromal cells (MSCs) display considerable benefit in the relief of GVHD through the deletion of alloactivated T cells. mAbs targeting cytokines or signaling molecules have been demonstrated to be potent therapeutic candidates for the prevention of GVHD. In this paper, we reviewed current advances in immunotherapy for GVHD.

## Immune Cells for the Treatment of GVHD

Donor T cells recognize alloantigens and are activated in GVHD process. The activation of dendritic cells (DCs) plays critical roles in the initiation of GVHD. Targeting immune cells, such as Tregs, as well as TDCs or mesenchymal stromal cells (MSCs) display considerable benefit in reducing GVHD through the deletion of alloactivated T cells. Coating allogeneic T cells showed significantly improved survival rate and relief GVHD. Moreover, the coating allogeneic T cells did keep still the effect of graft-versus-leukemia (GVL) (Table 1).

**Table 1:** Immune cells and monoclonal antibodies (mAbs) to cytokines for the treatment of graft-versus-host disease (GVHDs).

Different classes	Cells or agents	Mechanisms	Types of GVHD	Clinical translation
Immune cells	Regulatory T cells	Suppress the functions of T cells, natural killer (NK) cells, B cells, and APCs	aGVHD and cGVHD	Phase I clinical trials
	Tolerogenic dendritic cells	Modulate cytokines secretion, expand Foxp3^+^ Treg, and suppress allo-CD4^+^ T cell proliferation	aGVHD	Preclinical animal study
	Mesenchymal stromal cells	Regulate immunity by interacting with innate immune cells and adaptive immune cells	aGVHD	Being used in clinic
	MSC^-ICOS-EGFP^	Induce CD4^+^ T cell apoptosis, suppress Th1 and Th17 polarization, and promote Th2 polarization	aGVHD	Preclinical animal study
	Coating donor T cells	Block the direct contact between donor T cells and host APCs	aGVHD	Preclinical animal study

mAbs to cytokines	Daclizumab (humanized IL-2Rα mAb)	Inhibit activated alloreactive T cells	Gastrointestinal and hepatic aGVHD, steroid-refractory aGVHD (SR-aGVHD)	Being used in clinic
	Basiliximab (IL-2Rα mAb)	Inhibit activated alloreactive T cells	aGVHD and cGVHD	Being used in clinic
	Inolimomab (IL-2Rα mAb)	Inhibit activated alloreactive T cells	SR-aGVHD	Being used in clinic
	Infliximab	Inhibit TNF-alpha signaling and functions of T cells, NK cells, B cells, and APCs	SR-aGVHD	Being used in clinic
	Etanercept	Inhibit TNF-alpha signaling and functions of T cells, NK cells, B cells and APCs	Skin and gut aGVHD	Being used in clinic

*aGVHD, acute graft-versus-host disease; APC, antigen-presenting cell; cGVHD, chronic graft-versus-host disease; mAb, monoclonal antibody*.

### Therapeutic Potential of Tregs for GVHD

**Figure 1:**
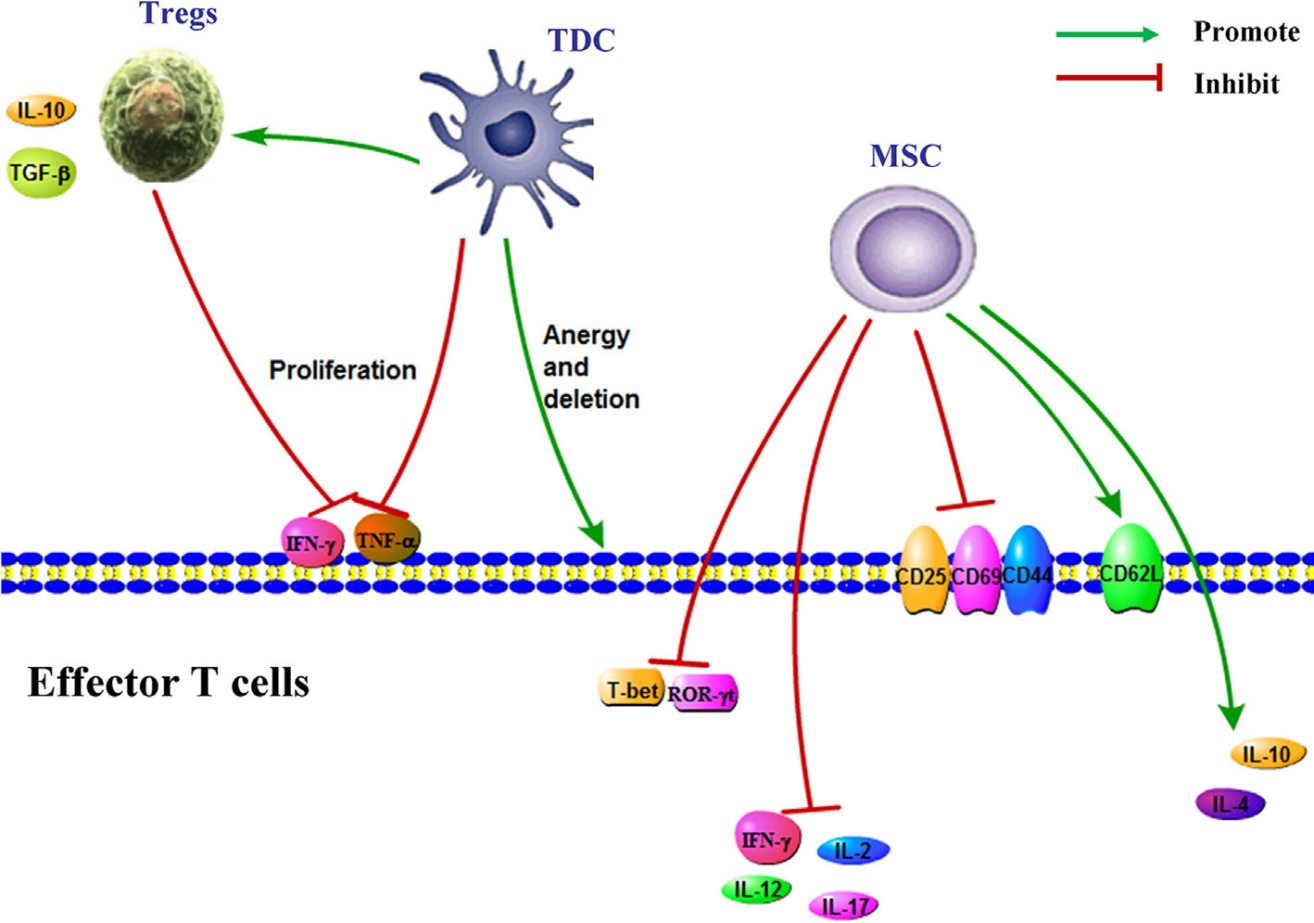
Immune cells for the treatment of Graft-versus-host disease. Regulatory T cells (Tregs) secret IL-10 and TGF-β and inhibit effector T cell proliferation and the production of IFN-γ and TNF-alpha. TDCs have low expression of MHC and co-stimulatory molecules, express high levels of immunosuppressive cytokines, expand Foxp3^+^ Treg and suppress allo-CD4^+^ T cell proliferation, and induce the anergy and deletion of effector T cell. MSC induce the expression of CD62L and the production of IL-4 and IL-10 in T cell and inhibit the expressions of ROR-γt, CD25, CD69, and CD44 and proinflammatory cytokines production. Abbreviations: MSC, mesenchymal stem cell; TDC, tolerogenic dendritic cells; Treg, regulatory T cell.

Host alloantigens are first recognized by donor T cells, and donor T cells become activated in GVHD process. An imbalance between Tregs and Th17 also involves in the pathological process. Tregs could suppress a wide range of cell types including B cells, T cells, APCs, and NK cells in MHC-unrestricted way. The roles for Tregs in inhibition of GVHD after HSCT have been demonstrated in both murine and human studies. As the negative regulators of immune responses to alloantigen, alloantigen-specific Tregs are critical for maintaining alloantigen-specific tolerance. Tregs strongly inhibited the division, expansion, and differentiation of donor T cells, suggesting a therapeutic potential of Tregs for GVHD. Tregs could protect from both acute and cGVHD (16). Tregs decrease the risk of allotransplant rejection through preventing autoimmune and allergic reactions (17, 18). Infusion of donor Tregs could prevent successfully aGVHD in mice and has shown promise in phase I clinical trials. Early Treg migration into lymphoid tissue and sustained donor Treg presence were important for GVHD prevention. Compared with control group, infusion of clinical-grade Tregs could also delay the onset of GVHD without causing any obvious toxicity or death in mice model (19) (Figure 1).

CCR8 could potentiate the survival of Tregs by promoting the interactions with DCs (20). Enforced IL-10 expression converts human CD4^+^ T cells into T regulatory type 1-like (CD4^IL-10^) cells that suppress effector T cells *in vitro* and mouse models (21). Short-lived apoptotic protein Fas ligand (FasL) increases the suppressive activity of Tregs and ameliorates GVHD severity (22). Pharmacological blockade or genetic deficiency of C3aR/C5aR signaling could augment the generation of induced Treg (iTreg), stabilize Foxp3 expression, and resist iTreg conversion to effector T cells producing IFN-γ/TNF-alpha, resulting in limiting GVHD (23). Natural Tregs might induce tolerance in allogeneic cell and organ transplantations. It was more efficient that alloantigen-specific Tregs controlled mice GVHD than that of polyclonal Tregs (24).

Clinical trials have been showed that Tregs had potential effects in preventing GVHD in patients undergoing allo-HSCT. Six independent trials showed the feasibility and safety of Treg-based approaches. Either freshly isolated or *ex vivo* expanded FOXP3^+^ Tregs were infused in patients undergoing allo-HSCT for onco-hematological diseases. Treg-treated patients, the cumulative incidence of relapse was significantly lower than in historical controls. The group of M. G. Roncarolo has completed a phase-I clinical trial in which IL-10-anergized T cells containing Tregs were injected in patients undergoing haploidentical-HSCT. Donor-derived IL-10-anergized T cells specific for host alloantigens were generated *in vitro* through activation of T cells by host-derived APCs in the presence of exogenous IL-10. M. G. Roncarolo demonstrated that no acute adverse events and only mild GVHD (grades II or III responsive to therapy) were observed after infusion of IL-10- anergized T cells (25, 26).

In addition to the role of CD4^+^ Tregs in suppressing excessive immune responses, CD8^+^ Tregs have also been reported to contribute in maintaining immune tolerance. Human alloantigen-specific CD8^hi^ Tregs have been generated in a large scale by Tu’s research group from University of Hong Kong. Tu’s research group demonstrated that *ex vivo-*induced CD8^hi^ Tregs controlled GVHD in an allospecific manner by reducing alloreactive T cell proliferation as well as decreasing inflammatory cytokine and chemokine secretion within target organs through a CTLA-4 dependent mechanism. These CD8^hi^ Tregs induced long-term tolerance effectively without compromising general immunity and graft-versus-tumor activity (27).

Martelli et al. reported that donor-derived Tregs, coinfused with conventional T cells (Tcons), could protect recipients against GVHD and prevent posttransplant leukemia relapse in phase II study (28). These findings demonstrate the immunosuppressive potential of Tregs in suppressing GVHD without loss of the benefits of graft-versus-leukemia (GVL) activity. It is very encouraging that the results of clinical trials applying Tregs in allo-HCT, which provides a basis for solid organ transplantation in future trials.

### Treatment of GVHD With TDCs

The activation of DCs from donor and recipient plays an important role in the initiation of GVHD. Donor T cells could be activated by host DCs alone. TDCs are essential for both peripheral and central tolerance (29). In thymus, TDCs take part in the autoreactive immature T cells deletion through presenting self-antigens. In periphery, the interactions between TDCs and T cells induce tolerance and the subsequent induction of Tregs, T cell deletion, and T cell anergy (30). TDCs express high levels of immunosuppressive cytokines and express low levels of MHC and co-stimulatory molecules. TDCs could reduce the severity of aGVHD by expanding Foxp3^+^ Treg, suppressing allo-CD4^+^ T cell proliferation and decreasing cytokines secretion. TDCs potently induce and maintain tolerance to a greater extent compared with conventional DCs. Donor or host TDCs promote allograft survival in mice. Furthermore, the levels of IL-10 and TGF-β in serum were significantly increased and the percentage of Foxp3^+^ cells continually elevated in the mice treated with TDCs (31) (Figure 1).

The use of TDC has shown great potential, and administration of TDC prolongs graft survival. Recipient DCs, donor DCs, or donor antigen-pulsed recipient DCs have been used in preclinical studies. Compared to immune inhibitor alone, autologous TDCs and suboptimal immune inhibitor combination are able to induce antigen specifical graft tolerance and long-term allograft survival. Similar TDCs in different animal models (mice and non-human primates) were derived, and the protective abilities of these TDCs were confirmed *in vitro* and *in vivo*. In rats, mice, and nonhuman primates, bone marrow progenitors cultured with low doses of granulocyte macrophage colony-stimulating factor could generate TDCs. Autologous TDCs are more effective than allogeneic TDCs in prolonging allograft survival. The mechanisms involved in the tolerance induced by autologous TDC might be that autologous TDC could specifically induce INF-gamma production by TCRαβ^+^CD3^+^CD4^−^CD8^−^ cells (double-negative T cells). It indicates the practical advantages of autologous TDCs as a therapeutic tool in clinic. This strategy may also help reduce the immunosuppressive load in grafted patients and, therefore, limit the harmful effects of immune inhibitors (32–34). The studies of TDCs in aGVHD treatment have being in preclinical study phase.

### Mesenchymal Stromal Cells for the Treatment of GVHDs

As a heterogeneous cell population, human multipotent mesenchymal stromal cells (MSCs) are present in many tissues and have immunomodulatory properties. MSCs downregulate immunity by interacting with innate immune cells [including macrophages, natural killer (NK) cells, and DCs], and adaptive immune cells (including B and T cells). MSCs have been clinically applied to treat autoimmune diseases and GVHD (35, 36). Since a 9-year-old boy with SR-aGVHD was first treated with haploidentical third-party derived MSCs, a number of clinical trial studies have suggested that MSC infusion might be effective and safe in aGVHD treatment (37). A phase II/III study using MSCs for grades II or III aGVHD was conducted by Muroi et al. 25 patients (grade IV, 3 patients and grade III, 22 patients) were enrolled and treated with MSC infusions. The rate of CR and partial response (PR) was 24% (6 patients) and 36% (9 patients), respectively, at 4 weeks after the first MSC infusions. And the adverse drug reaction commonly associated with MSC was not found. This result suggested that MSCs was effective for SR-aGVHD (38). The safety and feasibility of bone marrow-derived MSC was assessed in a phase I multicenter study with 40 patients. Overall response rate (ORR) was 67.5%, with 27.5% CR. The overall survival rate at 1 and 2 years was 50.0 and 38.6%, respectively, and the median survival time was 1.1 years from the first MSC administration (39). These findings show that MSC can be safely administered on top of conventional immunosuppression for SR-GVHD treatment.

Dotoli et al. reported that 46 patients received treatment with MSC infusion as salvage therapy for SR-aGVHD III/IV. The cumulative dose of MSCs was 6.81 × 10^6^/kg in a median of three infusions (range, 1–7). Result showed that 50% (23/46) presented clinical improvement all of the patients, The CR rate and PR rate was 13 and 61%, respectively. And 26% patients presented transient PR. 4.3% patients had acute side effects, such as blurred vision, vomiting, nausea, and cell infusion. These results show that this kind of therapeutic way is safe for SR-aGVHD (40). Placenta-derived decidual stromal cells (DSCs) are more immunosuppressive than MSCs and are used for aGVHD after HSCT as a novel therapy. Baygan et al. assessed the safety and adverse events of DSCs in 44 patients, and 40 controls were given. The result showed that 1-year survival rate was 67% for DSC treatment in GVHD patients, which was significantly better than control group (41). Jurado et al. reported adipose tissue-derived MSCs may be considered safe and feasible for cGVHD in combination with immunosuppressive therapy, which would likely have an impact on the course of cGVHD (42).

MSC ameliorated the pathological changes of liver and gut, and increased significantly survival in mouse aGVHD model. MSC therapy could directly inhibit the proliferation of donor CD4^+^ T cell and reduce the production of TNF-alpha (43). The beneficial effect of human MSCs was also associated with the alternation multiple aspects of mouse T cell activation. Moreover, the effects are specific to MSCs, non-MSC control cell lines were incubated with T cells, which had no any effects on the proliferation and activation of T cell (44). Bone marrow MSCs inhibit CD8^+^ T cell-mediated activation by decreasing the secretion of indoleamine 2, PGE2, TGF-β, and 3-dioxygenase and reducing the expression of natural-killer group 2, member D (NKG2D, activating/co-stimulatory receptor) (45) (Figure 1).

However, the safety of MSC has been focused attention on the possible malignant transformation due to mutations acquired during the large-scale expansion *in vitro*. A few studies described spontaneous oncogenic transformation in murine MSC (46). In order to investigate the frequency of cytogenetic alterations in a broad “collection” of clinical-grade BM-MSC products, Capelli et al. performed cytogenetic analysis of preparations expanded under Good Manufacturing Practice conditions. Their conclusion was that the presence frequency of spontaneous, non-clone, and non-recurrent mutations was not low, but the clone mutations obtained were not associated with a malignant transformation and transformed phenotype *in vitro* (47). Nevertheless, for safety reasons, the lack of clone chromosome aberrations or the presence of non-clone chromosome anomalies on 10% or less of metaphases were set as release criteria before MSC distribution for exploitation in clinical trials (48).

MSC^-ICOS-EGFP^ is a potent strategy for the prevention and treatment of aGVHD. MSC^-ICOS-EGFP^ could induce more the apoptosis of CD4^+^ T cell and suppress the polarization of Th17 and Th1, and promote Th2 polarization. In the MSC^-ICOS-EGFP^ treatment group, the levels of IL-4, IL-10 in serum were high, and the low levels of IL-2, IFN-γ, IL-12, IL-17A were found. MSC^-ICOS-EGFP^ could also induce the expression of STAT6, GATA-3 and inhibit STAT4, T-bet, ROR-γt expression (49). Despite substantial progress, how MSCs module immune responses during an aGVHD episode remains to be elucidated. The future studies of MSCs in aGVHD will lead to stepwise improvements in product selection, timing, dose, frequency, and method of administration. The optimization of MSC infusion therapy in aGVHD may supports the best use of MSC in other diseases of immunity and inflammation.

### Nanoencapsulation of Allogeneic T Cells Mitigates GVHD

**Figure 2:**
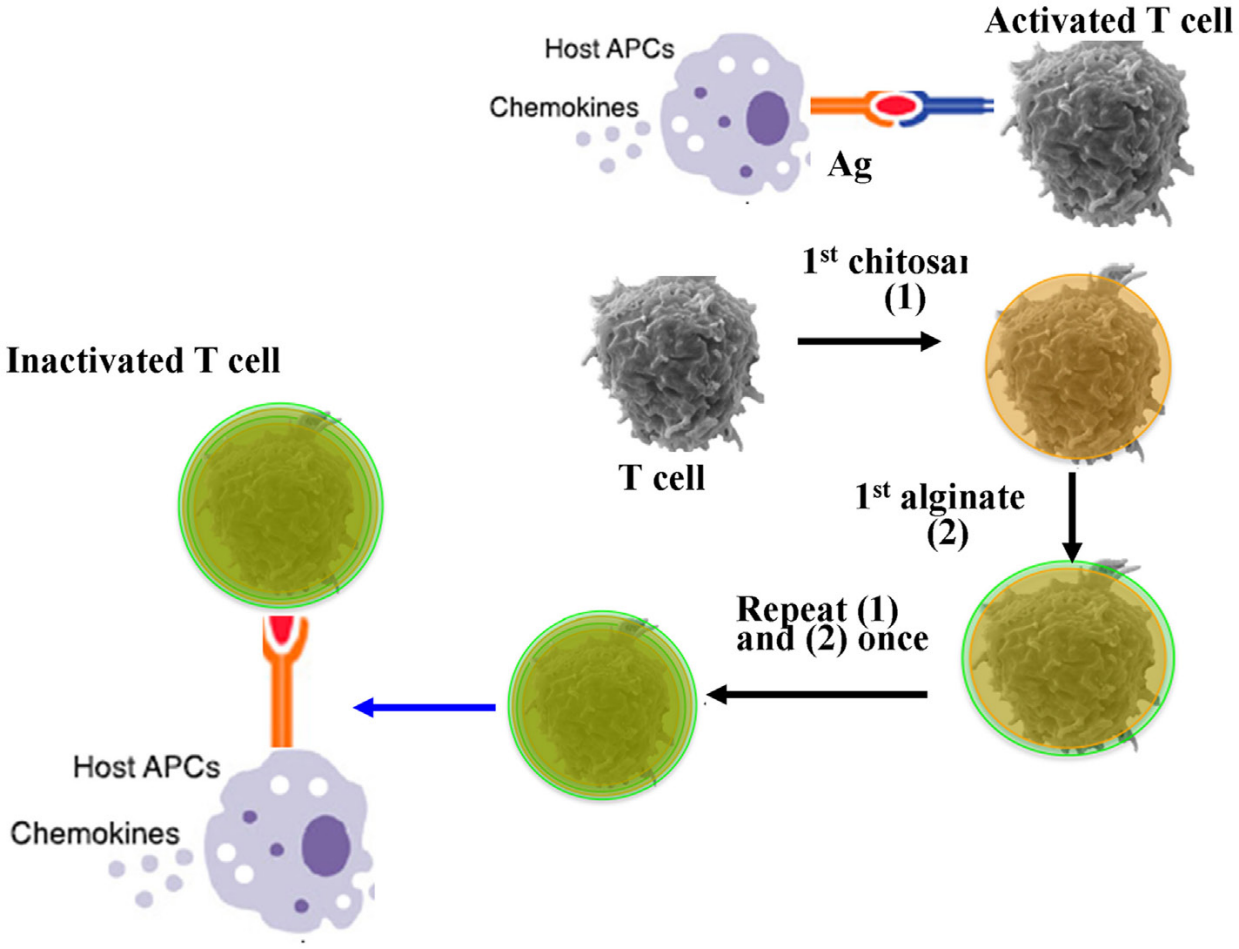
Nanoencapsulation of allogeneic T cells mitigate graft-versus-host disease (GVHD). Host antigen-presenting cells (APCs) activate donor T cells through presenting antigens. Donor T cells are coated with a nanoscale biocompatible and biodegradable film of chitosan and alginate. Encapsulated T cells could not receive the activated signals from host APCs, which could attenuate GVHD without compromising graft-versus-leukemia (52).

The activation of recipient APCs and donor T cells play key roles in the initiation progress of aGVHD. Therefore, the blockade of donor T cell activation by systemic immunosuppression is a common approach to combat aGVHD (50, 51). Coating donor T cells with nanoscale biocompatible and biodegradable film without significantly changing the size and surface charge of T cells is desired to block the direct contact between host APCs and donor T cells to minimize GVHD in allogeneic transplantation. In our lab, we tested if the temporary immunoisolation achieved by coating donor T cells with a biodegradable and biocompatible porous film of alginate and chitosan could relieve GVHD without compromising GVL effect. The results showed that nanoencapsulation had no impact on the phenotype of T cells *in vitro* in terms of viability, proliferation, size, cytokine secretion, and cytotoxicity effect against tumor cells. Lethally irradiated mice were transplanted with the encapsulated allogeneic T cells and bone marrow cells, the results exhibited that compared to the transplantation of nonencapsulated allogeneic T cells and BMCs, the survival was significantly improved and GVHD was reduced together with minimal liver damage and enhanced engraftment of donor BMCs. Moreover, the nanoencapsulation did not alter the GVL effect of encapsulated donor T cells (52). This finding suggest that nanoencapsulation of T cells with nanoscale, biodegradable, and biocompatible porous materials is a potentially effective and safe strategy to improve allogeneic HSC transplantation for hematological malignancies and other inflammatory and immune diseases (Figure 2).

## Targeting Cytokines for the Treatment of GVHD

Inflammatory cytokines secreted by activated T cells, macrophages, DCs, such as TNF-α and IL-2, and so on, are key inflammatory mediators of GVHD and may be important therapy targets. Various mAbs to cytokines secreted by effector cells in GVHD have been researched for aGVHD treatment. These mAbs include anti-TNF-α antibodies, IL-2 receptor antagonists, and so on (Table 1). Overall, the response rates are about greater than 60%, although the long-term survival still remains suboptimal (53).

### Interleukin-2 Receptor Antagonists and Ultra Low-Dose IL-2

The mAbs of IL-2 receptor targeting activated T cells have been investigated in SR-aGVHD treatment. Daclizumab is a humanized mAb against IL-2 receptor alpha subunit (IL-2Ra) and has been demonstrated to be effective and safe for adults refractory GVHD. Hamidieh et al. reported that daclizumab was given intravenously, and then was given again on a 10- to 14-day interval for maximum five times if necessary. The results showed that the long-term evaluation of daclizumab might be relatively safe and effective treatment in most of the severe SR pediatric patients with gastrointestinal aGVHD, although infection occurred frequently. 10 patients responded completely to daclizumab, only one patient responded partially to daclizumab, while there were remaining two patients failing to respond (54). 13 pediatric patients with refractory aGVHD were treated with daclizumab. After 30 days of daclizumab treatment, the CR rate and PR rate was 46 and 46%, respectively, in all patients, the CR rate and PR rate were both higher than that of control group. Moreover, the cutaneous aGVHD patients achieved CR. 50 and 30% had CR and PR, respectively, in gastrointestinal patients, whereas 11 and 55% of hepatic aGVHD patients reached CR and PR, respectively (55).

Combination treatment with infliximab and daclizumab is an effective therapy for SR-aGVHD patients and might be associated with decreased infection-related mortality compared to the monotherapy of infliximab or daclizumab (56). Seventeen GVHD received a combination anti-cytokine therapy of daclizumab and infliximab. This result suggested that the combination of anti-cytokine therapy of infliximab and daclizumab has significant activity in aGVHD (57). Basiliximab, another monoclonal antibody against IL-2Ra, prevents graft failure in renal transplantation and is also capable to effectively treat SR-GVHD. The side effects of daclizumab and basiliximab were tolerable and moderate. The prophylactic effects of daclizumab or basiliximab against GVHD in 82 peripheral blood stem cell transplantation patients were evaluated. The incidence rate of grades II–IV and III–IV aGVHD were 35.4 and 15.9%, respectively. 38.7% of evaluable patients suffered from cGVHD. Daclizumab or basiliximab could contribute to favorable outcome by preventing GVHD efficiently. Compared to daclizumab, basiliximab has superior activity against cGVHD, although has a similar effect on aGVHD (58, 59). Inolimomab (anti-IL-2Rα) is a mAb targeting IL-2 receptor subunit CD25 that predominantly inhibits activated alloreactive T cells. Several reports have showed encouraging results in SR-aGVHD treatment with inolimomab (60). But the combination of etanercept and inolimomab failed to improve the dismal prognosis of severe SR-aGVHD (61). Ultra low-dose (ULD) IL-2 could expand Tregs without diminishing antileukemic activity and antiviral activity in GVHD treatment. Tregs expressing high level of IL-2 receptor may selectively expand in response to low-dose IL-2 insufficient to stimulate effector T cell populations, thereby preventing GVHD (62).

### Anti-TNF Alpha mAbs

Infliximab is an anti-TNF alpha mAb that is effective for treatment of patients with SR-aGVHD (15, 63). However, some studies show controversial results. Couriel’s research group reported that 63 refractory aGVHD patients were included and randomized to receive either steroid therapy alone or steroid plus infliximab. Results showed that there were not significantly different between the two groups in GVHD-related mortality, non-relapse mortality, and overall survival (63). The possible explanation is that CD4^+^ Tregs express TNF receptor type 2 (TNFR2) and TNF-alpha could increase Treg activity through TNFR2. The sole defect of TNF production by donor T cells was sufficient to completely abolish the Treg suppressive effect in GVHD. Infliximab might reduce the inhibitory activity of Treg. The control of GVHD by Tregs was fully abolished by blocking TNFR2 or TNFR2-deficient Tregs (64).

Anti-TNF-alpha treatment enables to reduce corticosteroid dose without aggravating GVHD. Reduction of methylprednisolone and administration of infliximab could get rapid improvement of depression induced by steroid without aggravating GVHD (65).

Etanercept is a fusion protein of recombinant human soluble TNF-alpha receptor and has a modest effect on SR-aGVHD with tolerable side effects (66). 13 SR-aGVHD patients received etanercept therapy, six patients responded to etanercept, and the best response was seen in the patients with gastrointestinal aGVHD (67). The patients with grade I aGVHD receiving etanercept and topical corticosteroids showed a more decrease in the progression of grade than that of control group with corticosteroids alone (68). Etanercept had a down-grading effect on aGVHD, although no patient experienced a complete remission. In addition, gut and skin GVHD were also well controlled by etanercept, whereas hepatic GVHD was not the case (69).

## Targeting CD Molecules for the Treatment of GVHD

Effector cells involving in GVHD express many CD molecules, these CD molecules promote the differentiation, activation, maturation, and survival of effector cells. mAbs to CD molecules on cell surface have being investigated for GVHD treatment. These mAbs include anti-CD83, anti-CD132, anti-CD20, and anti-CD28 mAbs, and so on (Table 2), while anti-CD20 mAb has been extensively studied in cGVHD (53).

**Table 2:** Monoclonal antibodies (mAbs) to CDs and signaling molecules for the treatment of graft-versus-host disease (GVHD).

Different classes	Cells or agents	Mechanisms	Types of GVHD	Clinical translation
mAbs to CDs	sCD83 antibody	Inhibits DC-dependent cell proliferation and attenuate DC maturation	Prevent aGVHD in cardiac allograft	Preclinical animal study
	Rituximab (CD20 mAb)	Modulates cytokines secretion, expands Foxp3 + Treg, and suppresses allo-CD4+ T cell	Prevent cGVHD and preserve graft-versus-leukemia effect	Being used in clinic
	Anti-CD132 mAb	Inhibits granzyme B production in CD8+ T cells	Reverse liver and lung fibrosis in cGVHD	Phase II clinical trials
	Anti-CD45RC mAb	Induced rapid death of CD45RC high T cells through intrinsic cell signaling	Inhibited aGVHD in immune-humanized NSG mice	Preclinical animal study
	Anti-CD28 mAb	Suppress effector T cells, enhance regulatory T cells function and immune tolerance	Prevent aGVHD in mice	Preclinical animal study
	Anti-CD28 Fab antibody	Inhibits T cell expansion	Prevented aGVHD and cGVHD in mice	Preclinical animal study

Signaling molecules	DNMAML1	Blocks notch receptors and decreased Ras/MAPK and NF-κB activity	Decreased mortality and severity of aGVHD	Preclinical animal study
	R788 (inhibitor of Syk)	Downregulate the expressions of CXCR4, MCP-1, MIP-1alpha, IFN-gamma, IL-13, IL-17A	Attenuated the severity and fibrosis of cGVHD	Preclinical animal study
	PIAS3 [signal transducer and activator of transcription 3 inhibitor (STAT3)]	Blocks the IL-2-induced proliferation and provides selected immunosuppression	Attenuates the clinical and histopathological severities of aGHVD	Preclinical animal study
	KD025 (Rho-associated coiled-coil containing protein kinase2 inhibitor)	Inhibits the secretion of IL-21, IL-17, and IFN-γ, decreases phosphorylated STAT3	Suppresses murine and human cGVHD	Preclinical animal study
	Ruxolitinib (Janus kinases 1/2 inhibitor)	Impairs the differentiation of CD4+ T cells and increases FoxP3+ regulatory T cells	aGVHD and cGVHD	Multiple centers clinical trials

*aGVHD, acute graft-versus-host disease; APC, antigen-presenting cell; cGVHD, chronic graft-versus-host disease; DNMAML1, Dominant negative form of Mastermind-like 1; mAb, monoclonal antibody; UCBT, umbilical cord blood transplantation*.

### Soluble CD83 Molecules

CD83 belongs to immunoglobulin (Ig) superfamily and is a highly glycosylated type I transmembrane glycoprotein. As a marker of mature DCs, CD83 is expressed on DCs and activated lymphocytes and is essential for longevity of CD4^+^ T cells and thymus maturation. In addition, CD83 is also involved in the maturation, homeostasis, and function of peripheral B cell (70). Soluble CD83 (sCD83) may be derived from proteolytic cleavage of membrane-bound CD83. It has been demonstrated that sCD83 has therapeutic effects against GVHD by blocking CD83-ligand interaction in animal models. sCD83 is capable of inducing donor-specific allograft tolerance and attenuating DC maturation, and also inhibits the proliferation of DC-dependent allopeptide-specific T cell to prevent the rejections of cardiac allograft. Furthermore, sCD83 could also attenuate innate and adaptive immune responses, which results in preventing chronic rejection in a rat model with renal transplant (71, 72).

### Anti-CD83 Antibodies

It has been found that polyclonal or mAbs targeting CD83 can reduce GVHD symptoms through depleting activated CD4^+^ effector T cells and CD83^+^ DCs. In a human T cell-dependent peripheral blood mononuclear cell transplanted SCID (hu-SCID) model, CD83 antibody suppressed the alloproliferation of T lymphocytes but did not prevent engraftment of human T cells, including cytotoxic T lymphocytes (CTL) responsive to viruses and malignant cells. Polyclonal CD83 antibody for GVHD has been suggested to have the outcome of depletion of CD83^+^ DCs and mediates to suppress T cell proliferation. Anti-CD83 antibody treatment may leave tolerogenic and nonactivated CD83-tolerogenic DCs, which may induce Tregs with potential allo-suppressive benefits. Since activated CD4^+^ T cells also express CD83, anti-CD83 antibodies may also deplete the activated CD4^+^ effector T cells (73, 74). Therefore, administration of anti-CD83 antibodies may attenuate GVHD. Investigating the underlying mechanisms is likely to provide improved control of GVHD.

### Anti-CD20 Monoclonal Antibody

B cells involve in the pathogenesis of cGVHD. Rituximab, which is a monoclonal antibody of anti- CD20, has been investigated in cGVHD treatment and demonstrated to have some benefit. Cutler et al. reported that the response rate was 70% in 21 steroid-refractory cGVHD patients treated by rituximab (75). Similarly, the response rate to rituximab therapy was 66% in seven studies involving 111 patients by meta-analysis (76). Before the signs of cGVHD, administration of anti-CD20 mAb can prevent the induction of autoimmune-like cGVHD and preserve GVL effect; however, there is little effect if rituximab is administered after cGVHD onset (77).

### Anti-CD132 Monoclonal Antibody

CD132 is a subunit of the common gamma chain of the interleukin receptors for IL-2, IL-7, IL-9, IL-4, IL-21, and IL-15. The levels of these cytokines were shown to be high in aGVHD and cGVHD patients. Anti-CD132 monoclonal antibody could potently reduce aGVHD with respect to survival, GVHD histopathology, and the production of cytokines, such as TNF, IFN-γ, and IL-6. Anti-CD132 mAb afforded the protection from GVHD partly through inhibiting the production of granzyme B in CD8^+^ T cells. Also, T cells treated with anti-CD132 mAb displayed naive phenotype and showed decreased phosphorylation of JAK3. Additionally, anti-CD132 mAb reversed liver and lung fibrosis, and pulmonary dysfunction in the treatment of established cGVHD comparing with control group (78).

### Anti-CD45RC Monoclonal Antibody

CD45RC, a different isoform of CD45, plays an important role in thymocyte maturation and T cell activation and function. CD45RC is expressed at high levels on B cells, NK, and CD8^+^ T cells. CD4^+^ and CD8^+^ Foxp3^+^ Tregs do not express CD45RC and have strong immunoregulatory properties. Anti-CD45RC is a potent therapeutic candidate to induce transplantation tolerance in human. Anti-human CD45RC treatment inhibited GVHD in immune-humanized NSG mice. Administration of anti-CD45RC antibody could induce transplant tolerance associated with inhibition of allogeneic humoral responses in a rat cardiac allotransplantation model. Compared to control group, anti-CD45RC mAb induced rapid death of CD45RC^high^ T cells through intrinsic cell signaling, and preserved CD4^+^ and CD8^+^ CD45RC^low/−^ Tregs, which are able to adoptively transfer donor-specific tolerance to grafted recipients (79).

### Agonistic Anti-CD28 Monoclonal Antibody

Anti-CD28 monoclonal antibody targeting CD28 costimulatory molecule may be used as novel therapeutic agents to abrogate pathogenic T cell responses by selective depletion of activated T cells. Anti-CD28 antibodies differentiate from CTLA4Ig and cannot block CTLA-4 and PDL-1 coinhibitory signals. Anti-CD28 antibodies have the efficacies in suppressing effector T cells while enhancing Tregs function and immune tolerance. Administration of anti-CD28 mAb could inhibit donor T cell expansion and T cell costimulation and prevent GVHD in mice. Anasetti team found that anti-CD28 treatment prevented GVHD by selectively depleting alloantigen-activated donor T cells. Depletion of activated T cells mediated through CD28 did not depend on the expression of death receptors Fas, TNFRI, and TNFRII (80). FR104 is a novel humanized pegylated anti-CD28 Fab antibody fragment presenting a long elimination half-life in monkeys. *In vitro*, compared to control group, FR104 failed to induce human T cell proliferation and cytokines secretion, even in the presence of anti-CD3 antibodies. Administration of FR104 inhibited T cell expansion and prevented GVHD in humanized NOD/SCID mice in a CTLA4-dependent manner (81).

## Targeting Signaling Molecules for the Treatment of GVHD

Inflammatory cytokines, such as IL-2, TNF-α, and IL-17, and so on, mediate some signaling pathways through binding to receptor. These signaling pathways, such as Notch signaling pathway, Janus kinase (JAK)/STAT signaling pathway, participate in gene expression, cells interaction, cell proliferation, and activation, and so on in the pathological process of GVHD. Various inhibitors targeting key molecules in signaling pathways in GVHD have been investigated for GVHD treatment (Table 2).

### Notch Inhibitor for the Treatment of GVHD

**Figure 3:**
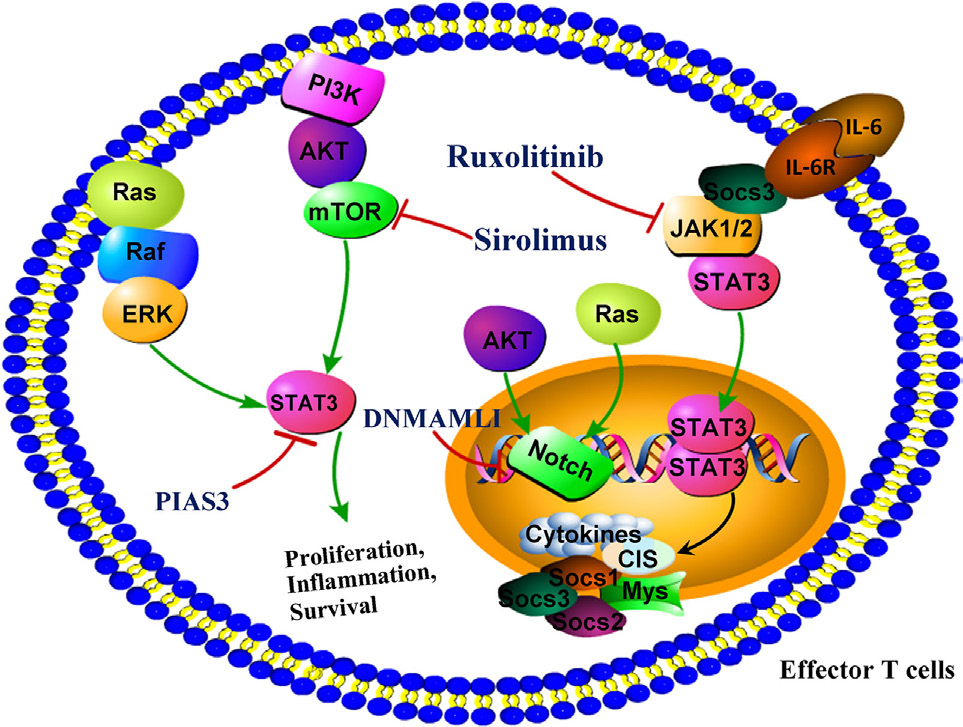
Targeting signaling molecules for the prevention of graft-versus-host disease (GVHD). DNMAML1 blocks the transcriptional activation downstream of all Notch receptors and reduces markedly GVHD severity. PIAS3, a protein inhibitor of activated signal transducer and activator of transcription 3 (STAT3), attenuated the clinical and histopathological severities of aGHVD. Ruxolitinib, which is a specific Janus kinase 1/2 inhibitor, represents a novel targeted approach in GVHD by suppression of proinflammatory signaling that mediates tissue damage. Sirolimus, a mTOR inhibitor, is effective in reducing incidence of GVHD after allo-HSCT. Abbreviations: DNMAML1, dominant negative form of Mastermind-like 1; mTORC, mammalian target of rapamycin complex; NOTCH, notch promoter; PIAS3, protein inhibitor of activated STAT3.

Notch signaling is a cell–cell communication pathway, which plays an important role in the development and immunity of T cell. Notch ligands bind to four Notch receptors (Notch1-4), which leads to the activation of proteolytic receptor. Dominant negative form of Mastermind-like 1 (DNMAML1) blocks the transcriptional activation downstream of all Notch receptors. DNMAML1 markedly decreased mortality of aGVHD in mice. DNMAML1 in donor T cells led to decreasing aGVHD severity markedly, without causing global immune suppression. Alloreactive T cells expressing DNMAMLI displayed increased expansion of Tregs and decreased production of inflammatory cytokines, leading to attenuating target organ damage (82) In addition, alloreactive T cells expressing DNMAMLI could also decrease the activation of Ras/MAPK and NF-κB signaling pathways (83) (Figure 3).

### Inhibition of c-Rel

NF-κB signaling plays important roles in immunity and oncogenesis and might be therapeutic targeting. c-Rel is one of NF-κB family members and a subunit of NF-κB. A novel strategy was developed that to ameliorate GVHD while preserving GVT activity by suppression of c-Rel (84). IT-901, which is a bioactive derivative of naphthalene thiobarbiturate, could potently suppress GVHD and preserve GVL effect though inhibiting c-Rel in allogeneic transplantation. The major mechanisms of IT-901 were to reduce alloactivation and impaire negative feedback on IL-2 production, resulting in the expansion of Tregs. Further preclinical assessment revealed the antitumor properties of IT-901 in the treatment of human B-cell lymphoma. This finding suggests that IT-901 is a novel therapeutic agent to ameliorate GVHD and treat lymphoid tumors (85).

### Syk Inhibitor

Syk, which is a protein tyrosine kinase, plays a key role in transmitting signals from receptors on cell surface. Syk phosphorylation increased in CD11b^+^ cells and lymphocytes during allogeneic transplantation. R788 is a potent inhibitor of Syk and attenuates the severity and fibrosis of cGVHD. The elevated expression of CXCR4 on T cells, B cells, and CD11b^+^ cells was significantly downregulated by R788. In addition, compared to control group, R788 inhibited the proliferation of CD11b^+^ cells and reduced mRNA expression levels of MCP-1, MIP-1alpha, IFN-gamma, IL-13, IL-17A, and TGF-beta1 in skin comparing with control group (86).

### Inhibitor of Signal Transducer and Activator of Transcription 3 (STAT3)

Signal transducer and activator of transcription 3 is a pivotal transcription factor for Th17 differentiation. The roles of STAT3 in cGVHD were demonstrated in mice model. Mice transplanted with inducible STAT3-deficient T cells had the same pulmonary function as healthy negative controls (87). PIAS3, a protein inhibitor of STAT3, inhibits STAT3 activation and significantly ameliorates the histopathology and clinical severities of aGHVD involving liver, lung, intestine, and skin comparing with control group. Inhibition of aGVHD by PIAS3 was largely associated to upregulating Th2 and Treg and downregulating Th17 and Th1 (88) (Figure 3).

KD025 is a selective inhibitor of Rho-associated coiled-coil containing protein kinase2 and effectively attenuates cGVHD in multiple animal models. Mice treated with KD025 resulted in normalization of pulmonary function, which resulted from a marked reduction of collagen deposition and antibody in lungs. Compared to control group, the frequency of T follicular helper cells decreased and T follicular regulatory cells increased in the spleens of mice treated with KD025, at the same time, STAT3 expression decreased. KD025 also inhibits the production of IL-17, IFN-γ, and IL-21, reduces protein expression of interferon regulatory factor 4 accompanied by decreasing phosphorylated STAT3 in peripheral blood mononuclear cells from the patients with active cGVHD (87).

### JAK 1/2 Inhibitor Improve Survival of Mice With aGVHD

The important roles of host inflammatory response governed by JAK 1/2 have been also highlighted through some novel insights into the pathology of aGVHD. Activated JAK 1/2 are required for T effector cell responses. Ruxolitinib is a specific JAK 1/2 inhibitor. The potent anti-inflammatory properties of ruxolitinib have been demonstrated by preclinical study. Ruxolitinib might be a novel potential approach for GVHD by suppression of tissue damage mediated by proinflammatory signaling. Ruxolitinib could increase FoxP3^+^ Tregs and impair the differentiation of CD4^+^ T cells into the cells producing IL17A and IFN-gamma (89, 90) (Figure 3).

Ruxolitinib was recently employed to treat SR-GVHD and a promising ORRs was found. In a retrospective survey, 95 SR-GVHD patients from 19 stem cell transplant centers in the United States and Europe received ruxolitinib as salvage therapy. Results showed that the ORR in SR-aGVHD was 81.5%, and the ORR in SR-cGVHD was 85.4%. The 6-month survival was 79 and 97.4% for SR-aGVHD and SR-cGVHD, respectively. The ORR and survival rate were both higher than control group. However, the adverse effects were observed in both SR-aGVHD and SR-cGVHD patients during ruxolitinib treatment, such as the reactivation of cytomegalovirus and cytopenia (91). Khandelwal et al. reported that ruxolitinib was less efficacy in children HSCT patients with a high rate of reversible adverse effects in children with SR-aGVHD (92).

## Conclusion

The physiopathology of GVHD is complicated involving a variety of immune cells and molecules. The treatment and management of GVHD may be proposed, trialed, and ultimately validated. Although there is good evidence supporting treatment of both aGVHD and cGVHD with steroids, clearly there remains an unmet clinical need to develop novel safe therapeutic approaches. Some potential therapy strategies are being found, in particular, the roles of Tregs, TDCs, coating T cell, and MSC in GVHD treatment. IL-2Ra, anti-TNF alpha monoclonal antibody, targeting CD molecules and signaling molecules have been demonstrated to be effective for GVHD. The effects of Tregs against GVHD are very encouraging, but Tregs might also diminish the GVL effects mediated by NK or T cell. Despite substantial progress, how MSCs module immune responses when administered peripherally during an aGVHD episode remains to be elucidated. However, these therapy strategies remain to be validated in clinic. In addition, individualized therapy should be considered based on the characterization of GVHD pathophysiological mechanisms involved, stages, and subtypes. It is anticipated that the novel therapeutic targets and promising strategies may improve the outcomes of GVHD in the future.

## Author Contributions

LZ wrote the paper. JY collected data, and WW revised and wrote the paper.

## Conflict of Interest Statement

The authors declare that the research was conducted in the absence of any commercial or financial relationships that could be construed as a potential conflict of interest.
